# A Supervised Learning Approach for Accurate and Efficient Identification of Chikungunya Virus Lineages and Signature Mutations

**DOI:** 10.3390/biology14121736

**Published:** 2025-12-04

**Authors:** Miao Miao, Yameng Fan, Jiao Tan, Xiaobin Hu, Yonghong Ma, Guangdi Li, Ke Men

**Affiliations:** 1 School of Public Health, Xi’an Medical University, Xi’an 710021, China; miaomiao@xiyi.edu.cn (M.M.);; 2 Institute of Epidemiology and Health Statistics, School of Public Health, Lanzhou University, Lanzhou 730000, China; huxiaobin@lzu.edu.cn; 3 Hunan Provincial Key Laboratory of Clinical Epidemiology, Xiangya School of Public Health, Central South University, Changsha 410078, China; liguangdi@csu.edu.cn

**Keywords:** Chikungunya virus, genotyping, machine learning, SHAP, signature mutations

## Abstract

Chikungunya virus (CHIKV) poses a significant public health threat, and its continuous evolution necessitates high-resolution genomic surveillance. Current methods lack the speed and resolution to efficiently discriminate sub-lineages. To address this, we developed CHIKVGenotyper, an interpretable machine learning framework for high-resolution CHIKV lineage classification. This study leveraged a comprehensive dataset of 6886 CHIKV genome sequences, from which a high-quality set of 3014 sequences was established for model development. A hierarchical assignment pipeline that integrated a probability-based sequence matching model, machine learning refinement, and phylogenetic validation was developed to assign high-confidence labels across eight CHIKV lineages, thereby constructing a reliable dataset for subsequent analysis. Multiple machine learning models were trained and evaluated, with the optimal Random Forest model achieving near-perfect accuracy (F1-score: 99.53%) on high-coverage whole-genome test data and maintaining robust performance (F1-score: 96.50%) on an independent low-coverage set. The E2 glycoprotein alone yielded comparable accuracy (F1-score: 99.52%), highlighting its discriminative power. SHapley Additive exPlanations (SHAP) analysis identified key lineage-defining amino acid mutations, such as E1-K211E and E2-V264A, for the Indian Ocean Lineage, which were corroborated by established biological knowledge. This work provides an accurate, scalable, and interpretable tool for CHIKV molecular epidemiology, offering insights into viral evolution and aiding outbreak response.

## Simple Summary

The chikungunya virus causes a painful disease and continues to spread and evolve, making global monitoring of new outbreaks very important. However, current methods for tracking different lineages of the virus are often slow and not precise enough. To solve this, we created a new approach that can rapidly and accurately identify specific viral lineages from their full genetic sequence. We developed our model using a large dataset of thousands of viral genomes and showed that it achieves exceptional accuracy (99.53%). Remarkably, the tool maintained high performance even when analyzing only a specific part of the virus, demonstrating a potential pathway for efficient monitoring. Our approach not only classifies the virus but also identifies the key genetic changes that define each lineage, some of which we confirmed to match existing biological knowledge. This work provides scientists and public health officials with a fast, reliable, and easy-to-understand tool to track the virus’s spread, understand how it is evolving, and better respond to future outbreaks.

## 1. Introduction

Chikungunya fever (CHIKF) is an arboviral disease caused by the chikungunya virus (CHIKV). Its clinical manifestations range from mild febrile illness and arthralgia during acute infection to more severe chronic conditions such as polyarthralgia, polyarthritis, and rheumatism, making it a resurgent threat to public health [1,2,3,4,5]. The virus is transmitted to humans through the bites of infected female *Aedes* mosquitoes, primarily *Aedes aegypti* and *Aedes albopictus* [6,7]. As an enveloped, positive-sense RNA virus belonging to the genus *Alphavirus* (family *Togaviridae*) [8], CHIKV has a genome of approximately 11.8 kb that encodes two open reading frames (ORFs) flanked by 5′ and 3′ untranslated regions (UTRs). These ORFs give rise to four non-structural proteins (nsP1–nsP4) and five structural proteins (C, E3, E2, 6K, E1) [9]. Based on phylogenetic relationships, CHIKV is categorized into several major genotypes, including the East/Central/South African (ECSA), West African (WA), Asian, and the Indian Ocean Lineage (IOL) [10,11,12]. Emerging from the ECSA genotype, the IOL has been responsible for large-scale outbreaks across the Indian Ocean islands, South and Southeast Asia, and parts of Europe [13,14], and has become the dominant circulating genotype globally in recent years.

The high mutability of CHIKV, attributable to its error-prone RNA-dependent RNA polymerase, facilitates the emergence of adaptive mutations that can enhance viral fitness, alter transmission dynamics, or expand geographic range [15]. Historically, CHIKV transmission was mainly mediated by *Aedes aegypti* [16]. However, adaptive mutations in the E1 and E2 envelope glycoproteins of the ECSA-IOL strain have enhanced its ability to infect and be transmitted by *Aedes albopictus* [17,18]. In the presence of the E1-A226V substitution, mutations in non-structural proteins augment viral fitness in *Aedes albopictus* and *vertebrates* [19,20]. More recently, IOL strains lacking E1-A226V have been identified carrying novel combinations of mutations such as E1-K211E and E2-V264A, which have been responsible for major outbreaks since 2014 [21,22,23,24]. Subsequent acquisitions, including E1-I317V, have further promoted the widespread dissemination of this sub-lineage [9]. Other mutations, such as those in E2 (e.g., L210Q, K252Q, K200R, I211T), have also been implicated in enhanced viral transmission and pathogenicity [25,26,27,28]. In addition, research indicates that two specific mutations (Q192L and C483Y) in nsP4 are associated with reduced sensitivity to the antiviral drug 4′-Fluorouridine [29]. These continuous genetic adaptations highlight the importance of genomic surveillance in tracking CHIKV evolution and informing public health responses [30,31].

Despite the clear need for timely and high-resolution genotyping, current methods for CHIKV lineage classification remain limited in several aspects. First, the resolution of existing tools, such as ArboTyping (http://krisp.org.za/tools.php, accessed on 10 August 2025), is often restricted to distinguishing the three major genotypes (ECSA, WA, Asian), failing to discriminate clinically and epidemiologically important sub-lineages like IOL [32]. Finer-grained classification still heavily depends on constructing phylogenetic trees, which is a computationally intensive, time-consuming, and impractical process for the rapid analysis of large-scale genomic data [33,34]. Second, there is a lack of automated frameworks capable of systematically identifying and interpreting key amino acid mutations that define lineage-specific signatures. This limits the ability to gain biological insights into the drivers of CHIKV evolution and spread. Therefore, the development of a computational tool that enables rapid, high-resolution lineage classification while automatically pinpointing critical signature mutations is of paramount importance for advancing CHIKV molecular epidemiology and surveillance.

**Figure 1:**
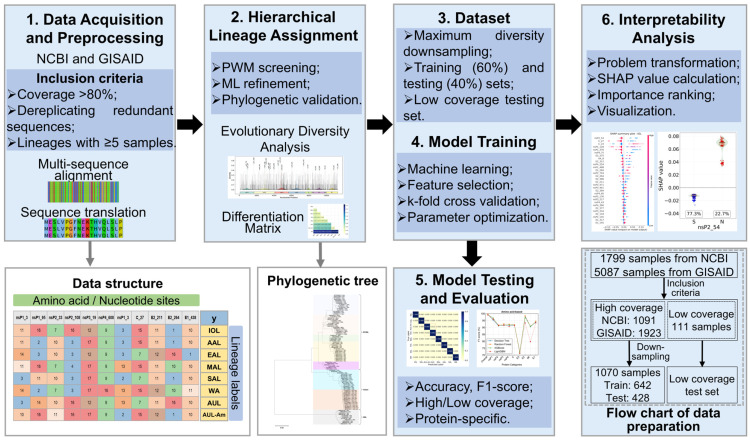
Processing pipeline for CHIKV lineage classification and signature mutation identification.

To address these challenges, we developed an integrated machine learning framework (CHIKVGenotyper) for high-resolution CHIKV lineage identification and mutation analysis. Machine learning has demonstrated considerable success in addressing similar bioinformatic challenges, enabling rapid variant classification for viruses [35,36], early warning of concerning variants [37], and accurate cross-species genotyping of structural variations [38]. Inspired by these advances, our study integrates a hierarchical lineage assignment strategy, robust machine learning modeling, and model interpretability techniques to achieve accurate, scalable, and biologically interpretable genotyping, as outlined in Figure 1. The main contributions of this work are as follows:

(1) We constructed a high-quality CHIKV genome dataset with fine-grained lineage labels using a hierarchical classification pipeline that combines rapid Position Weight Matrix (PWM) screening, targeted machine learning refinement, and phylogenetic validation.
(2) We developed and evaluated multiple machine learning models for discriminating eight CHIKV lineages, achieving near-perfect accuracy on high-coverage whole-genome data and maintaining robust performance on low-coverage sequences.
(3) We employed SHapley Additive exPlanations (SHAP), an interpretability framework, to identify and validate key amino acid substitutions associated with specific lineages, thereby bridging data-driven predictions with established biological knowledge and offering novel insights into CHIKV evolution and adaptation.

This comprehensive framework provides a practical and efficient tool for genomic surveillance, outbreak investigation, and molecular epidemiology research.

## 2. Materials and Methods

### 2.1. Data Collection and Preprocessing

The dataset for this study integrated CHIKV complete genome sequences collected from two databases up to 10 August 2025. A total of 1799 sequences were obtained from the National Center for Biotechnology Information (NCBI) database, of which 1099 sequences were annotated with fine-grained lineage labels via Nextstrain (https://nextstrain.org/), covering nine lineages: South American Lineage (SAL), Eastern African Lineage (EAL), African/Asian Lineage (AAL), Asian Urban Lineage (AUL), AUL–America (AUL-Am), IOL, WA, and Sister Taxa to ECSA (sECSA). Additionally, 5087 sequences were obtained from the Global Initiative on Sharing All Influenza Data (GISAID) database, annotated with three main genotypes: ECSA, WA, and Asian.

A systematic preprocessing pipeline was implemented to construct a high-quality dataset for machine learning. First, multiple sequence alignment was performed against the reference genome (NCBI Accession: MN974211.1) using MAFFT version 7.490. The aligned sequences were then trimmed to remove the 5′ and 3′ untranslated regions. Subsequently, rigorous quality control was applied: (1) removing sequences with <80% coverage; (2) dereplicating redundant sequences; and (3) excluding lineages with fewer than five samples (e.g., sECSA) to avoid overfitting. This process resulted in a high-coverage dataset of 3014 sequences for model training, optimization, testing, and interpretability analysis. Additionally, 111 sequences with 80–90% coverage were retained as an independent low-coverage test set to evaluate model generalization on suboptimal data.

### 2.2. Evolutionary Diversity Analysis

To understand the genetic variation characteristics of CHIKV and provide input features for subsequent machine learning model construction, evolutionary diversity analysis at the nucleotide and amino acid levels was performed on the preprocessed high-coverage dataset, using amino acid sequence diversity as an example. To quantify the variation degree at each amino acid site, a site diversity index ($D_{p}$) was introduced. For a given amino acid site p, let $c_{i}$ be the count of amino acid type ${at}_{i}$, and $N_{p}$ be the total number of effective sequences (gaps excluded). The frequency $f_{i}$ of amino acid ${at}_{i}$ is calculated as $f_{i}=c_{i}/N_{p}$. The diversity index ($D_{p}$) for this site is defined as follows:

(1) $$D_{p}=1-\underset{i}{\max}\left(f_{i}\right)$$

Based on this index, a site was defined as non-conserved ($D_{p}$ > 0) upon detection of any sequence polymorphism. This permissive threshold guaranteed a comprehensive dataset for the downstream hierarchical lineage assignment. Additionally, to further quantify the evolutionary divergence among different lineages, a lineage differentiation matrix was calculated using the method described in our previous work [39], and the result was visualized as a heatmap.

### 2.3. Hierarchical Lineage Assignment for Unlabeled Samples

To enable rapid and accurate lineage assignment of unlabeled samples, we developed a hierarchical pipeline that integrates probability-based sequence matching with machine learning classifiers, reserving phylogenetic analysis for low-confidence cases.

#### 2.3.1. Construction of a Representative Reference Sequence Set

To establish a robust benchmark for subsequent phylogenetic validation, a high-quality reference set ($R_{ref}$) for the eight known lineages was established from the NCBI high-coverage sequences. To ensure diversity and minimize bias, sequences with ≥95% coverage were selected. For lineages with large sample sizes, we applied a Maximum Diversity Downsampling (MDD) approach. This greedy algorithm iteratively selected the sequence with the largest average nucleotide difference from those already chosen, thereby maximizing the representation of genetic diversity within each lineage.

#### 2.3.2. Development of Complementary Classification Models

Two collaborative models were developed to address lineage classification at different resolutions.

(1) Position Weight Matrix (PWM) Model

A PWM was constructed for each lineage from the NCBI high-coverage dataset to capture its characteristic sequence patterns. The process was as follows:

For lineage k with M aligned sequences, non-conserved sites were selected according to the definition in Section 2.2 ($D_{p}$ > 0), yielding a set of L sites for PWM construction. For each site j, the frequency of each nucleotide (A, C, G, T) was calculated with a pseudo-count α (set to 1) to avoid zero probabilities:

(2) $$P_{j}^{k}\left(X\right)=\frac{{count}_{j}\left(X\right)+\alpha }{M+4\alpha }$$

where ${count}_{j}\left(X\right)$ is the number of occurrences of nucleotide X at site j.

This frequency was converted to a log-odds score relative to the genomic background frequency $b_{X}$. Therefore, the score $S_{j}^{k}\left(X\right)$ assigned by the PWM to nucleotide X at site j is as follows:

(3) $$S_{j}^{k}\left(X\right)=\log_{2}\left(\frac{P_{j}^{k}\left(X\right)}{b_{X}}\right)$$

The resulting PWM for each lineage is a 4×L matrix of these scores. For a query sequence Q, the matching score to lineage k was calculated as follows:

(4) $$\xi_{k}=\sum_{j=1}^{L}S_{j}^{k}\left(Q_{j}\right)$$

where sites with gaps or ambiguous characters contributed zero.

The scores for all eight lineages were normalized into a probability distribution using the softmax function [40]. Classification confidence ($c_{1}$) was defined as the difference between the highest and second-highest probabilities. Initial analysis indicated that the PWM model struggled to distinguish three genetically similar lineage pairs: [AUL, AUL-Am], [IOL, EAL], and [AAL, MAL].

(2) Machine Learning (ML) Models

To resolve the above ambiguities, three independent binary Random Forest classifiers were trained, one for each confusing lineage pair. Prior to model training, feature dimensionality reduction from the pool of non-conserved sites was performed to select the most discriminative nucleotide sites, as detailed in Section 2.4.

The models were trained on samples from the NCBI high-coverage dataset with optimization via 5-fold cross-validation. The classifiers’ prediction probabilities [41] were used to define the ML confidence score ($c_{2}$).

#### 2.3.3. Hierarchical Lineage Assignment Workflow

The lineage assignment of unlabeled samples proceeded through the following steps:

PWM Screening: The sample was initially classified using the PWM model. If $c_{1}$ exceeded a confidence threshold ($c_{1}^{th}$) and the assigned lineage was not one of the three ambiguous pairs (e.g., WA or SAL), the result was accepted.

ML Refinement: If the PWM model assigned a lineage from one of the ambiguous pairs, the corresponding binary Random Forest classifier was activated. The ML result was accepted only if both $c_{1}$ exceeded $c_{1}^{th}$ and $c_{2}$ exceeded its threshold ($c_{2}^{th}$).

Phylogenetic Validation: Samples that remained “ambiguous” after the above steps were analyzed phylogenetically [32]. A phylogenetic tree was constructed using MEGA 11, incorporating the ambiguous sample and the $R_{ref}$ set, applying both Maximum Likelihood and Neighbor-Joining methods. The lineage was confirmed only if both phylogenetic methods yielded a consistent conclusion; otherwise, the sample was labeled “uncertain”.

This hierarchical strategy enables rapid automatic genotyping for most samples while ensuring the reliability of genotyping for ambiguous samples through the “gold standard” of phylogenetic analysis, providing a high-quality dataset for subsequent research.

### 2.4. Construction of High-Precision Lineage Identification Models

Following the assembly of a high-quality dataset with precisely assigned lineage labels, we developed and systematically evaluated multiple machine learning models for high-accuracy CHIKV lineage classification.

#### 2.4.1. Dataset Partitioning and Balancing

The NCBI and GISAID high-coverage datasets were merged (*n* = 3014). To address class imbalance, we applied the MDD strategy from Section 2.3, capping each lineage at 200 samples to create a balanced dataset (*n* = 1070). This dataset was then randomly divided into a training set ($S_{train}$, *n* = 642) and an independent test set ($S_{test}^{h}$, *n* = 428) in a 6:4 ratio, with stratification by lineage. A separate low-coverage test set ($S_{test}^{l}$, *n* = 111) was held out for robustness evaluation.

#### 2.4.2. Feature Dimensionality Reduction and Key Site Selection

To reduce dimensionality and select discriminative lineage markers, feature importance analysis was conducted. The analysis considered two feature sets, comprising non-conserved nucleotide sites for nucleotide-based models and the corresponding amino acid sites for amino acid-based models.

Preliminary classification models were trained on $S_{train}$ using four algorithms: Decision Tree (DT), Random Forest (RF), XGBoost (XGB), and LightGBM (LGB). After optimizing core parameters via 5-fold cross-validation, each model was retrained on $S_{train}$ to assign an importance weight to each feature. To integrate the advantages of all four algorithms, a conservative selection strategy was adopted: the final feature subset was defined as the union of all sites with importance weights greater than zero in every model. This process yielded 1197 key nucleotide sites and 591 key amino acid sites.

#### 2.4.3. Model Training and Performance Evaluation

Final models were trained on the selected feature sets using $S_{train}$, with separate models for nucleotide and amino acid features. Each model underwent another round of hyperparameter optimization via 5-fold cross-validation. Performance was evaluated on the independent test sets $S_{test}^{h}$ and $S_{test}^{l}$. To objectively assess multi-class performance and account for potential class imbalance in the test data, the weighted F1-score was adopted as the primary evaluation metric. As the harmonic mean of precision and recall, the weighted F1-score provides a balanced and representative measure of overall model performance across lineages. Additionally, the Receiver Operating Characteristic (ROC) curve and the area under the curve (AUC) were analyzed to comprehensively evaluate the models’ classification performance [42,43].

By comparing the weighted F1-scores, accuracy, and confusion matrices of each model, the best-performing nucleotide-based and amino acid-based lineage identification models were selected. Additionally, a protein-specific model comparison was performed by training and testing classifiers on features from individual viral proteins to assess the relative contribution of each genomic region to classification performance. The optimal model from the full-genome analysis was subsequently used for global interpretability analysis.

### 2.5. Model Interpretability Analysis

To uncover the molecular basis of CHIKV lineage discrimination, we employed explainable machine learning on the optimal amino acid-based model, as amino acid substitutions most directly reflect biological function. The SHAP framework was utilized to quantify feature importance, providing a mathematically robust interpretation of model predictions. The workflow consisted of the following steps:

(1) The multi-class lineage classification problem was decomposed into binary tasks. For each target lineage, labels were binarized into “target lineage” versus “other lineages”. A separate classifier was trained for each binary task on the amino acid feature training set.
(2) For each binary model, SHAP values were computed for every amino acid feature site across training samples. The sign of a SHAP value indicates whether a specific amino acid promotes (positive) or suppresses (negative) prediction toward the target lineage, while its magnitude reflects the degree of the influence. Sites were then ranked in descending order based on their mean absolute SHAP value.
(3) SHAP summary plots were generated to visualize the overall impact and contribution direction of the most important sites. Feature dependence plots were generated to illustrate how different amino acid types at individual key sites influence the SHAP values for a given lineage.

## 3. Results

### 3.1. Landscape of Genetic Diversity Across CHIKV Genomic Regions

To systematically evaluate the genetic variation in CHIKV, we calculated the diversity index for each nucleotide and amino acid site across its coding regions. The analysis encompassed four non-structural proteins (nsP1, nsP2, nsP3, nsP4) and five structural proteins (C, E3, E2, 6K, E1), revealing distinct patterns of conservation and polymorphism across the viral genome. The summary statistics for both nucleotide and amino acid diversity are consolidated in Table 1.

**Figure 2:**
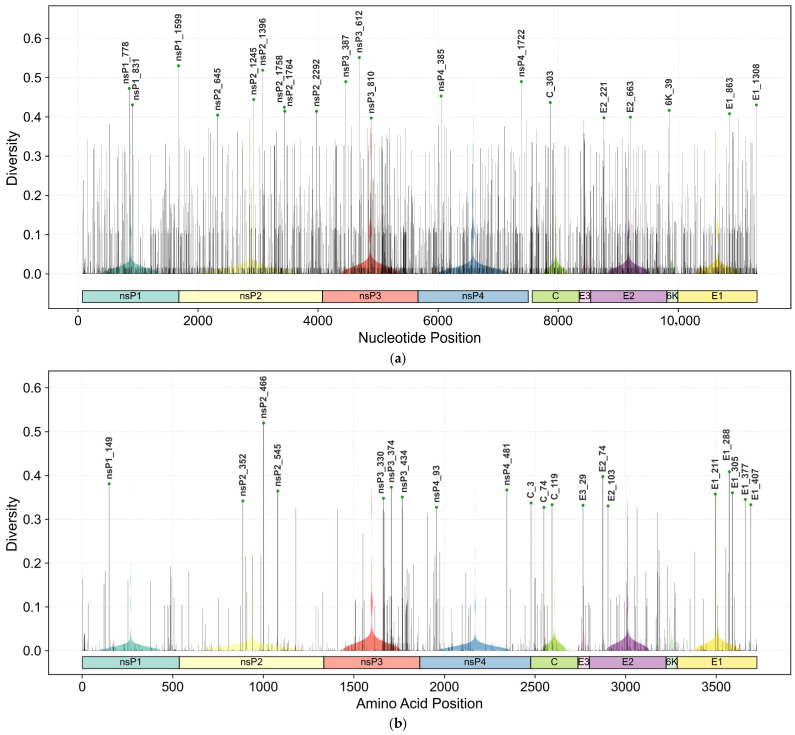
Genetic diversity landscape of the CHIKV genome. (**a**) Nucleotide diversity distribution; (**b**) amino acid diversity distribution. High-diversity sites (top 20) are annotated with genomic positions. Protein regions are color-coded in the horizontal bars below the plots. Violin plots show the distribution of diversity values within each protein region.

Based on the statistical analysis, nsP3, E3, E2, and 6K exhibit consistently higher diversity compared to other proteins at both nucleotide and amino acid levels. Notably, nsP3 displays the highest proportion of polymorphic amino acid sites (73.53%), followed by 6K (73.33%) and E2 (71.80%). Furthermore, Figure 2 visually summarizes the genetic diversity landscape of CHIKV, depicting the nucleotide and amino acid diversity distributions across the viral genome. High-diversity sites are highlighted and annotated with genomic positions. Protein regions are demarcated by color-coded bars below the diversity plot. Violin plots superimposed on the diversity profile illustrate the distribution of diversity values within each protein region.

**Figure 3:**
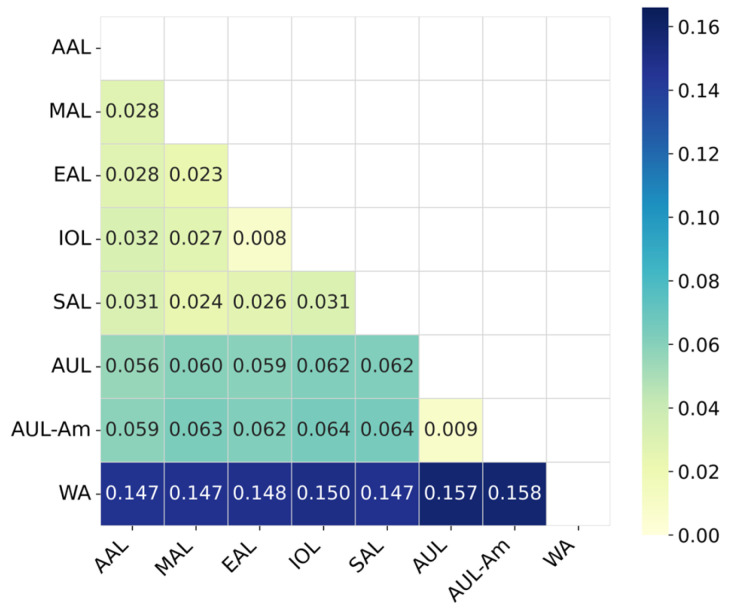
Differentiation matrix of the eight CHIKV lineages. Each element in the matrix represents the degree of evolutionary divergence between two corresponding lineages, with darker colors indicating greater differentiation.

To further elucidate the evolutionary relationships among the defined lineages, the genetic differentiation matrix is presented in Figure 3. This matrix quantitatively confirms substantial divergence of the WA lineage from all others (differentiation > 0.14). Conversely, the minimal differentiation observed between IOL and EAL, as well as AUL and AUL-Am (differentiation < 0.01), underscores the close genetic relationship within these pairs, thereby explaining the greater challenge in their accurate classification.

### 3.2. Hierarchical Lineage Assignment

**Figure 4:**
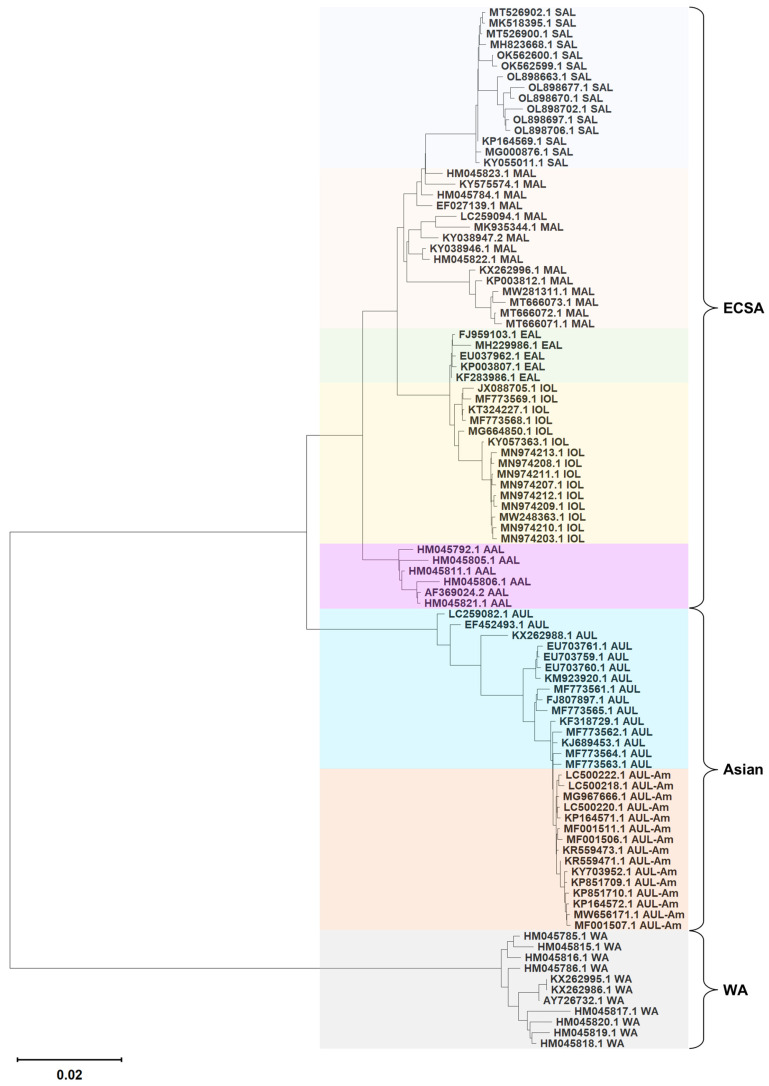
Phylogenetic tree of the representative reference sequences for the eight CHIKV lineages. The tree was constructed using the Maximum Likelihood method in MEGA 11. A maximum of 15 sequences per lineage were included. Lineage names are indicated following the NCBI accession numbers. Major genotypes (ECSA, Asian, WA) are annotated on the tree, and lineages are highlighted with distinct color backgrounds.

To establish a robust dataset for model development and achieve accurate lineage classification, we implemented a hierarchical lineage assignment pipeline. The representative reference sequence set ($R_{ref}$) constructed for phylogenetic validation exhibited clear evolutionary relationships among the eight lineages, as shown in the phylogenetic tree provided in Figure 4.

The PWM model achieved an initial screening accuracy of 95.78% on the high-coverage NCBI dataset with known labels. The corresponding confusion matrix (Figure S1) confirmed the model’s high overall performance while revealing persistent confusion between three specific lineage pairs: [AUL, AUL-Am], [IOL, EAL], and [AAL, MAL]. This observation directly motivated the development of targeted machine learning classifiers for these ambiguous cases. For samples that remained unclassified after both PWM and ML steps, phylogenetic analysis using the reference set provided definitive lineage assignment, with two representative examples illustrated in Figure S2. Besides the two AAL samples misclassified as MAL shown in Figure S2b, six AUL-Am samples initially assigned as AUL were also identified and correctly reclassified through phylogenetic validation.

### 3.3. Performance of High-Precision Identification Models

**Figure 5:**
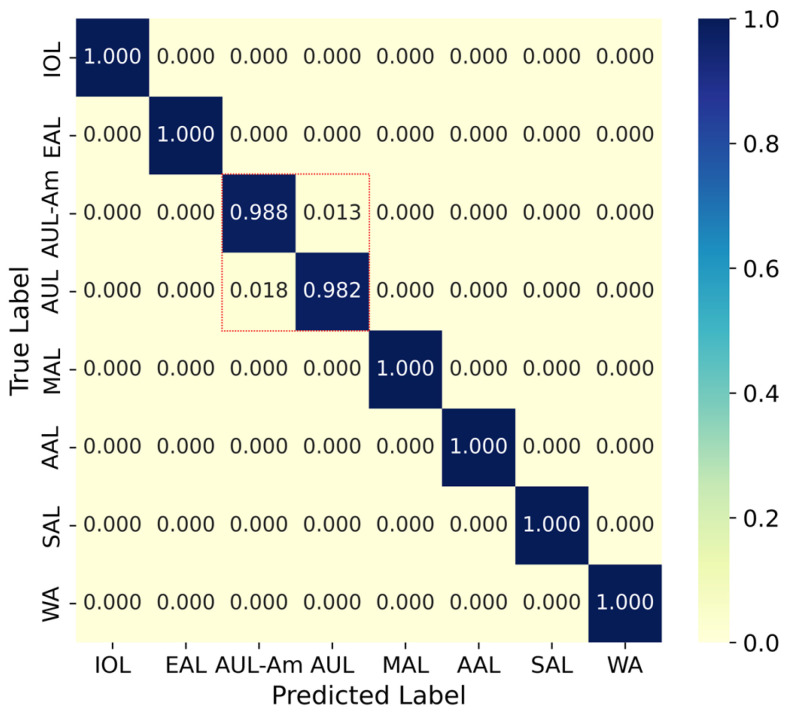
Confusion matrix of the optimal Random Forest classifier using nucleotide-based features on the high-coverage test set ($S_{test}^{h}$). The model demonstrates robust discriminatory capability across all eight CHIKV lineages, with misclassification confined exclusively to the evolutionary proximal AUL and AUL-Am pair, which is highlighted by a red dashed box.

Comprehensive evaluation on the high-coverage test set ($S_{test}^{h}$) using whole-genome features demonstrated outstanding performance across most machine learning methods (Table 2). The corresponding ROC curves for both nucleotide-based (Figure S3) and amino acid-based (Figure S4) models are provided, with AUC values explicitly annotated. The Random Forest classifier achieved the best performance on both nucleotide-based and amino acid-based feature sets, attaining a top F1-score of 99.53%. Notably, the Random Forest model exhibited virtually identical classification efficacy with both feature types. Three methods (Random Forest, LightGBM, and Decision Tree) achieved F1-scores exceeding 99%, with only the XGBoost model (nucleotide-based) falling slightly below this threshold. Figure 5 presents the confusion matrix of the optimal Random Forest model utilizing nucleotide-based features on the high-coverage test set. The results confirm exceptional classification accuracy across all lineages, with only minor misclassification observed exclusively between the closely related AUL and AUL-Am lineages.

Model performance on the independent low-coverage test set ($S_{test}^{l}$) is summarized in Table S1, with the corresponding ROC curves for both nucleotide-based (Figure S5) and amino acid-based (Figure S6) models provided. Consistent with the findings on high-coverage data, the Random Forest classifier achieved the highest F1-scores on both nucleotide-based (95.12%) and amino acid-based (96.50%) feature sets, demonstrating remarkable generalization capability to incomplete sequence data. Notably, the Decision Tree model performed substantially better with amino acid features (F1-score: 95.10%) than with nucleotide features (F1-score: 89.82%), whereas LightGBM showed the opposite trend. Figure S7 displays the confusion matrix for the optimal Random Forest model utilizing amino acid-based features on the $S_{test}^{l}$. It is important to note that the $S_{test}^{l}$ dataset contained no samples from EAL and AAL. The model correctly classified the majority of low-coverage sequences, with misclassification confined to a subset (30%) of AUL-Am samples being assigned to AUL.

**Figure 6:**
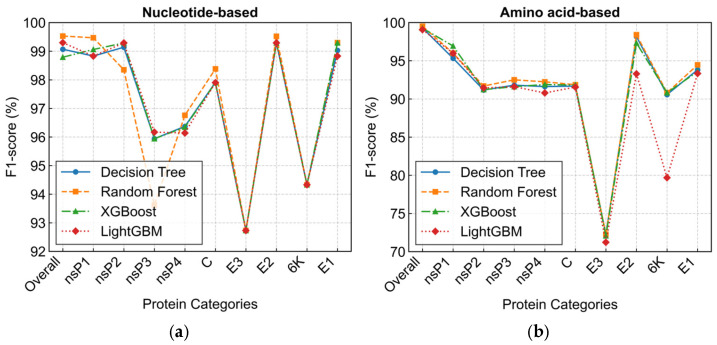
Performance comparison of lineage classification models trained on features from individual viral proteins. The weighted F1-scores were evaluated on the high-coverage test set ($S_{test}^{h}$). (**a**) Models built on nucleotide-based features; (**b**) models built on amino acid-based features. The whole-genome model performance is included as a benchmark.

To identify genomic regions with high discriminatory power for lineage identification, we evaluated models trained on features from individual viral proteins using the high-coverage dataset. As shown in Figure 6, models utilizing nucleotide-based features (Figure 6a) outperformed their amino acid-based counterparts (Figure 6b) across most genomic regions. Several protein regions, specifically nsP1, nsP2, E2, and E1, achieved near-whole-genome classification accuracy when using nucleotide features. The E2 glycoprotein gene was particularly discriminative, with the Random Forest model attaining an F1-score of 99.52%, virtually matching the best whole-genome performance (99.53%). Similarly, for amino acid-based classification, three methods (Random Forest, Decision Tree, and XGBoost) achieved their highest performance on the E2 glycoprotein, with Random Forest performing best (F1-score = 98.41%). A complementary visualization of model performance in terms of accuracy, which corroborates these findings, is provided in Figure S8.

### 3.4. Identification of Signature Mutations from SHAP Analysis

**Figure 7:**
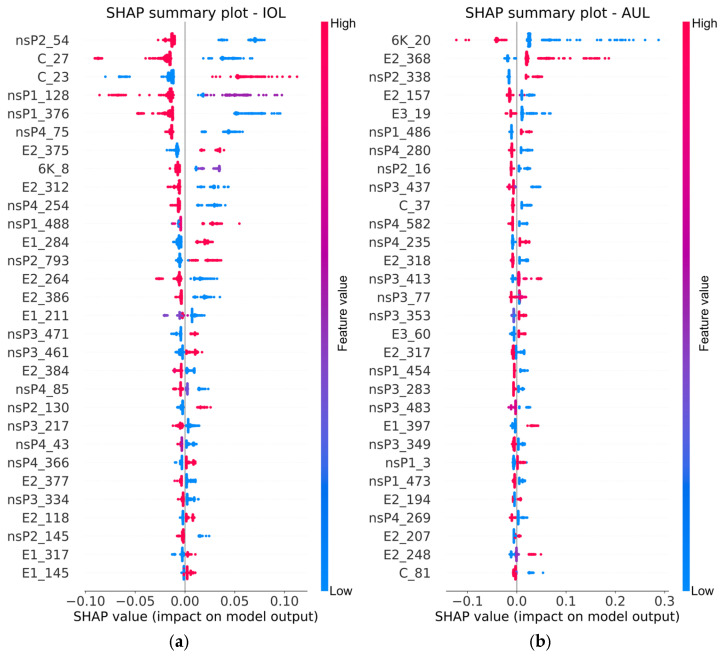
SHAP summary plots for IOL and AUL. The plots visualize the top 30 features ranked by the mean absolute SHAP value for (**a**) IOL and (**b**) AUL. Each point represents a sample. The feature’s impact on the model output is shown on the x-axis (SHAP value), and the features are ordered on the y-axis by importance. The color represents the amino acid identity (encoded as an integer) at that site for each sample. The SHAP value indicates the feature’s impact on the prediction, and a positive SHAP value increases the probability of the sample being classified as the target lineage.

To elucidate the molecular determinants of CHIKV lineage classification, we performed model interpretability analysis using SHAP on the optimal Random Forest model trained with amino acid-based features. Figure 7 presents the SHAP summary plots for IOL and AUL, representative of the ECSA and Asian genotypes, respectively, displaying the top 30 amino acid sites ranked by their mean absolute SHAP values. The summary plots for the remaining six lineages are provided in Figure S9.

In these plots, each point represents a single sample. The horizontal position indicates the SHAP value, which quantifies the impact of that amino acid feature on the prediction for the target lineage. A positive SHAP value increases the probability of the sample being classified as the target lineage, while a negative value decreases it. The vertical axis lists the features, ordered from most to least important. For IOL, sites such as nsP2_54, C_27, E2_264 and E1_211 emerged as positive drivers for its classification. For AUL, sites including 6K_20, E2_368, nsP2_338, E3_19 and C_37 were identified as key discriminators.

**Figure 8:**
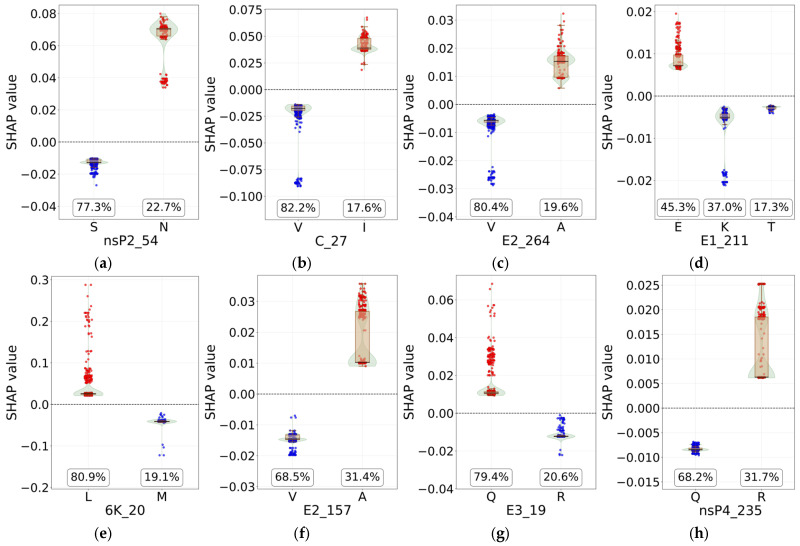
SHAP dependence plots for key discriminatory amino acid sites in IOL and AUL. The top row (**a**–**d**) shows four critical sites for IOL, and the bottom row (**e**–**h**) shows four for AUL. For each site, the x-axis indicates the amino acid type, the y-axis shows the SHAP value (with a dashed line at zero), individual samples are colored by their SHAP value (blue: negative, red: positive), and distributions for each amino acid are summarized with boxplots and violin plots.

To further dissect the specific impact of amino acid substitutions, we generated SHAP dependence plots for representative sites. Figure 8 shows four sites for IOL (top row) and four for AUL (bottom row). In these plots, the x-axis denotes the amino acid type (and its prevalence in the dataset), the y-axis shows the corresponding SHAP value, and a dashed line marks a SHAP value of zero. Individual samples are plotted as points, colored blue (negative SHAP value) or red (positive SHAP value). The distribution of SHAP values for each amino acid type is further detailed by overlaid boxplots and violin plots. As shown in Figure 8d, the presence of Glutamate (E) at site E1_211 strongly promotes IOL classification, while Lysine (K) and Threonine (T) at the same site suppress IOL prediction. Similarly, Figure 8f demonstrates that Alanine (A) at site E2_157 contributes positively to AUL identification, whereas Valine (V) at this position exhibits a negative effect. The corresponding dependence plots for the other six lineages are provided in Figure S10.

## 4. Discussion

This study presents a comprehensive framework for high-resolution lineage classification of CHIKV, integrating hierarchical assignment, machine learning, and model interpretability to achieve accurate and scalable genotyping. Our work addresses a critical gap in current surveillance efforts, which often rely on low-resolution genotyping or computationally intensive phylogenetic analysis. The development of models capable of identifying eight distinct CHIKV lineages with near-perfect accuracy represents a significant advancement for molecular epidemiology and outbreak tracking.

A major innovation of our approach lies in the hierarchical lineage assignment pipeline for unlabeled samples, which strategically combines efficiency and accuracy. Whereas automated lineage designation tools like Autolin [44] focus on extracting lineage structures from large phylogenies, our pipeline is designed for the efficient and precise classification of individual sequences into pre-established, biologically defined lineages. This fundamental difference makes our tool particularly suited for rapid screening and real-time surveillance, where speed and assignment to known lineages are critical. The initial PWM screening achieved a high accuracy of 95.78%, demonstrating its utility for rapid, large-scale pre-classification. However, its limitation in resolving three closely related lineage pairs ([AUL, AUL-Am], [IOL, EAL], and [AAL, MAL]) necessitated a secondary, targeted ML refinement step. This hierarchical design proved crucial, as final phylogenetic validation of low-confidence samples identified and corrected misclassifications. Notably, two GISAID-derived samples (EPI_ISL_17456024 and EPI_ISL_18490232) originally assigned as MAL were definitively reclassified as AAL through phylogenetic analysis. This underscores the indispensable role of the phylogeny-based “gold standard” in ensuring final label integrity, especially for resolving subtle evolutionary relationships, a challenge also acknowledged in the development of the ArboTyping tool [32], which utilizes a similar BLAST-to-phylogeny workflow for robust genotype calls.

Existing classification methods, such as the one implemented in the ArboTyping tool [32], typically resolve CHIKV sequences to the level of the three major genotypes (ECSA, WA, Asian). Consequently, they lack the resolution to distinguish the eight finer sub-lineages, such as the clinically and ecologically important IOL, which is crucial for detailed outbreak tracing. Finer resolution currently remains heavily dependent on constructing phylogenetic trees. In contrast, our method provides this finer resolution automatically, achieving 100% accuracy at the genotype level while successfully discriminating sub-lineages with F1-scores exceeding 99.5% on high-coverage data. Compared to other machine learning-based genotyping approaches [34,39], CHIKVGenotyper provides interpretable identification of key mutations via SHAP analysis and employs a hierarchical assignment pipeline robust to limited sample sizes. Moreover, CHIKVGenotyper operates with high efficiency, classifying a single sample in under 10 milliseconds without the need for computationally expensive phylogenetic tree inference.

The misclassification between the evolutionarily proximal AUL and AUL-Am pair is most likely due to the lower genetic divergence resulting from their phylogenetic relationship, where AUL-Am constitutes a sub-clade within AUL, consistent with Figure 3 and Figure 4 and prior findings [45,46]. Impressively, the models maintained robust performance on an independent low-coverage test set (F1-score up to 96.50%), demonstrating strong generalization ability to suboptimal data, a common scenario in real-world surveillance. It should be noted that this test set did not include EAL and AAL samples due to data scarcity; thus, performance on these specific lineages under low coverage requires future validation with external datasets. Nonetheless, the near-perfect accuracy achieved by all four methods on the high-coverage test set, coupled with Random Forest’s consistent generalization to the low-coverage scenario for the other lineages, underscores the model’s substantial utility.

The systematic evaluation of protein-specific classification models provided valuable insights into the genomic determinants of lineage identity. This analysis revealed that models trained on nucleotide or amino acid features from single proteins, particularly the E2 glycoprotein, could achieve classification accuracy approaching whole-genome levels. The E2 glycoprotein emerged as the most discriminative region, with the Random Forest model attaining an F1-score of 99.52% using nucleotide features. This finding is biologically plausible, as the E2 protein is a major target of host immune pressure and a key mediator of cell entry, driving adaptive evolution [15,47]. The high classification accuracy based on E2 alone underscores its predominant role in lineage definition and highlights the robustness of our method. It demonstrates that accurate lineage identification can be achieved even with specific genomic segments, reducing sequencing requirements and facilitating the use of our tool in resource-limited settings or for historical datasets where only partial genomes are available [48].

Another cornerstone of this study is the application of explainable SHAP to decipher the molecular basis of the model’s decisions, translating complex model predictions into biologically intelligible rules. The SHAP analysis pinpointed key amino acid substitutions across structural and non-structural proteins that serve as hallmark signatures for specific lineages. For instance, for the IOL, critically positively associated sites included nsP2_54, C_27, E2_264, and E1_211. The identification of E1-K211E and E2-V264A as key discriminators for IOL is strongly supported by prior research [49]. These mutations, which arise on an E1-V226A background, have been experimentally shown to enhance CHIKV fitness in *Aedes aegypti* mosquitoes, facilitating a vector shift that contributed to outbreaks in the Indian subcontinent and Southeast Asia, including the 2021 Malaysian outbreak [15,49]. Additionally, nsP2-E145D and nsP4-S55N mutations have been reported as defining features of the Indian subcontinent/Southeast Asia clade within the IOL [9,49]. Our model independently and quantitatively identified these mutations as drivers for IOL classification, validating their biological relevance. Furthermore, for the EAL, a key discriminant was identified at nsP1_171. The presence of glutamine (Q) at this site (nsP1-R171Q) has been linked to enhanced pathogenicity in primate cells [20], providing a potential phenotypic correlate for this lineage-specific signal. Similarly, for the AUL, sites such as E2_157 and E3_19 were highlighted. The concordance between our data-driven SHAP results and experimentally verified mutations from the literature indicates that our model identifies features strongly associated with CHIKV lineage diversification, thereby enhancing the credibility and interpretability of its predictions.

Despite its strengths, this study has limitations. The hierarchical pipeline, while accurate, still requires phylogenetic validation for a small subset of ambiguous samples. The persistence of low-confidence predictions, particularly between AUL and AUL-Am, may indicate the emergence of novel sub-variants that are not well-captured by the current lineage definitions. Additionally, our feature selection was based on aligned sequences. Exploring alignment-free methods for lineage classification could further enhance the pipeline’s speed, scalability, and applicability. Finally, the continuous evolution of CHIKV necessitates periodic retraining of the models with updated sequence data to maintain their classification accuracy over time.

## 5. Conclusions

In this study, we developed and validated an accurate and interpretable machine learning framework for high-resolution CHIKV lineage classification. The hierarchical assignment pipeline enabled the construction of a high-quality, lineage-annotated dataset, which supported the training of robust classification models. The top-performing Random Forest classifier achieved an F1-score of 99.53% on high-coverage whole-genome data and maintained strong performance, with an F1-score of 96.50%, on an independent low-coverage test set, demonstrating excellent generalization capability. Furthermore, we showed that models trained solely on E2 glycoprotein features could approach whole-genome accuracy, attaining an F1-score of 99.52%, underscoring both the biological relevance of this region and the potential for resource-efficient lineage identification. By integrating hierarchical lineage assignment, robust modeling, and explainable machine learning, this work provides a practical tool for genomic surveillance, outbreak investigation, and molecular epidemiology research. The identification of key signature mutations through SHAP analysis, including E1-K211E and E2-V264A for the IOL, offers biologically interpretable insights into CHIKV evolution and adaptation, effectively bridging data-driven predictions with established experimental knowledge.

## Figures and Tables

**Table 1:** Summary of nucleotide and amino acid diversity across CHIKV protein-coding regions.

Protein	Location (nts)	Protein Length	Nucleotide Diversity		Amino Acid Diversity	
			Polymorphic Sites (Ratio)	Median (Q1, Q3)	Polymorphic Sites (Ratio)	Median (Q1, Q3)
nsP1	77–1681	534	974 (60.69%)	0.0006 (0, 0.0024)	330 (61.80%)	0.0003 (0, 0.0012)
nsP2	1682–4075	797	1398 (58.40%)	0.0003 (0, 0.0021)	433 (54.33%)	0.0003 (0, 0.0009)
nsP3	4076–5665	529	1141 (71.76%)	0.0009 (0, 0.0124)	389 (73.53%)	0.0009 (0, 0.0018)
nsP4	5666–7501	611	1101 (59.97%)	0.0003 (0, 0.0033)	340 (55.65%)	0.0003 (0, 0.0009)
C	7567–8349	260	445 (56.83%)	0.0003 (0, 0.0024)	140 (53.85%)	0.0003 (0, 0.0012)
E3	8350–8541	63	126 (65.63%)	0.0009 (0, 0.0136)	41 (65.08%)	0.0009 (0, 0.0030)
E2	8542–9810	422	852 (67.14%)	0.0009 (0, 0.0100)	303 (71.80%)	0.0009(0, 0.0021)
6K	9811–9993	60	124 (67.76%)	0.0009 (0, 0.0036)	44 (73.33%)	0.0009 (0, 0.0021)
E1	9994–11,313	439	848 (64.24%)	0.0009 (0, 0.0031)	288 (65.60%)	0.0006 (0, 0.0012)

**Table 2:** Model performance on the high-coverage test set $S_{test}^{h}$.

Method	Evaluation Metrics	Feature	
		Nucleotide-Based	Amino Acid-Based
Decision Tree	Precision (%)	99.10	99.32
	Recall (%)	99.07	99.30
	F1-score (%)	99.07	99.29
	AUC	0.9960	0.9960
Random Forest	Precision (%)	99.53	99.54
	Recall (%)	99.53	99.53
	F1-score (%)	99.53	99.52
	AUC	1.0000	1.0000
XGBoost	Precision (%)	98.85	99.31
	Recall (%)	98.83	99.30
	F1-score (%)	98.79	99.26
	AUC	1.0000	0.9999
LightGBM	Precision (%)	99.32	99.07
	Recall (%)	99.30	99.07
	F1-score (%)	99.30	99.06
	AUC	0.9999	0.9999

## Data Availability

Codes and models are available at https://github.com/MiaoMiaoXiYi/CHIKVGenotyper (accessed on 23 October 2025). Data were obtained from the Global Initiative on Sharing All Individual Data (GISAID) (https://www.gisaid.org/ (accessed on 10 August 2025)) and the National Center for Biotechnology Information (NCBI) (https://www.ncbi.nlm.nih.gov/ (accessed on 10 August 2025)).

## Supplementary Materials

The following supporting information can be downloaded at https://www.mdpi.com/article/10.3390/biology14121736/s1. Figure S1: Confusion matrix of the initial lineage screening using the Position Weight Matrix (PWM) model on the high-coverage NCBI dataset; Figure S2: Phylogenetic validation of ambiguous samples with two representative cases; Figure S3: ROC curves of the nucleotide-based models on the high-coverage test set; Figure S4: ROC curves of the amino acid-based models on the high-coverage test set; Figure S5: ROC curves of the nucleotide-based models on the low-coverage test set; Figure S6: ROC curves of the amino acid-based models on the low-coverage test set; Figure S7: Confusion matrix of the optimal Random Forest classifier using amino acid-based features on the low-coverage test set; Figure S8: Performance comparison of lineage classification models trained on features from individual viral proteins in terms of accuracy; Figure S9: SHAP summary plots for six CHIKV lineages; Figure S10: SHAP dependence plots for key discriminatory sites in six lineages; Table S1: Model performance on the low-coverage test set.

## Abbreviations

The following abbreviations are used in this manuscript:

CHIKV	Chikungunya virus
SHAP	SHapley Additive exPlanations
CHIKF	Chikungunya fever
ORFs	Open reading frames
UTRs	Untranslated regions
nsP	Non-structural proteins
ECSA	East/Central/South African
WA	West African
IOL	Indian Ocean lineage
PWM	Position weight matrix
NCBI	National Center for Biotechnology Information
GISAID	Global Initiative on Sharing All Influenza Data
SAL	South American lineage
EAL	Eastern African lineage
AAL	African/Asian lineage
AUL	Asian Urban lineage
AUL-Am	AUL–America
sECSA	Sister Taxa to ECSA
MDD	Maximum diversity downsampling
ML	Machine learning
DT	Decision tree
RF	Random forest
XGB	XGBoost
LGB	LightGBM
ROC	Receiver operating characteristic
AUC	Area under the curve
