# Supplementary material for: A Supervised Learning Approach for Accurate and Efficient Identification of Chikungunya Virus Lineages and Signature Mutations
Source: Biology (Basel). 2025 Dec 4;14(12):1736. doi: 10.3390/biology14121736 (PMC12730473; doi:10.3390/biology14121736)
Supplement: Supplementary file 1 [file biology-14-01736-s001.zip › biology-3981015-supplementary.pdf]

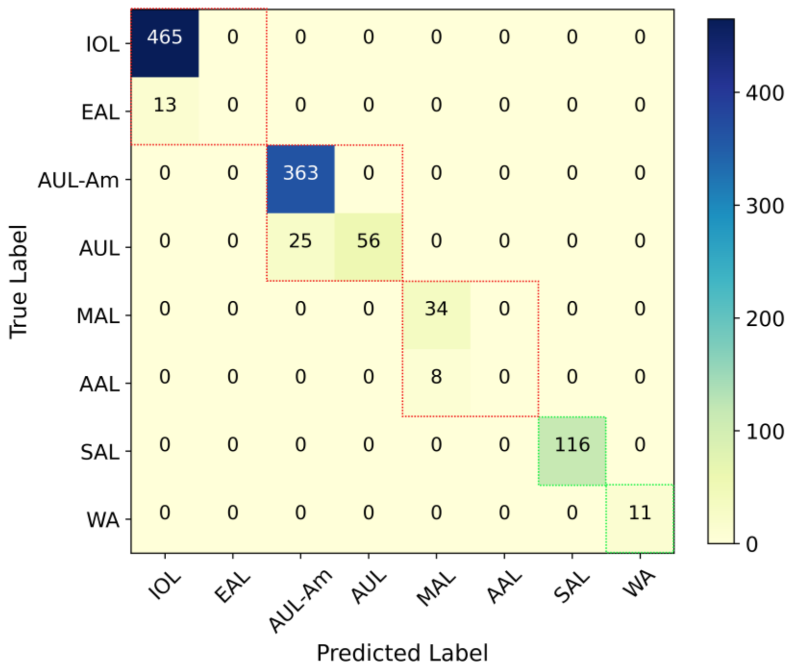

Figure S1. Confusion matrix of the initial lineage screening using the Position Weight Matrix (PWM) model on the high-coverage NCBI dataset. The matrix displays the number of samples, with the model's predictions on the x-axis and the true lineage labels on the y-axis. The PWM model achieved an overall accuracy of 95.78%. Three lineage pairs that were frequently confused by the PWM model are highlighted by red dashed boxes, justifying the subsequent development of targeted machine learning classifiers. In contrast, the WA and SAL lineages, which achieved 100% classification accuracy, are indicated by green dashed boxes.

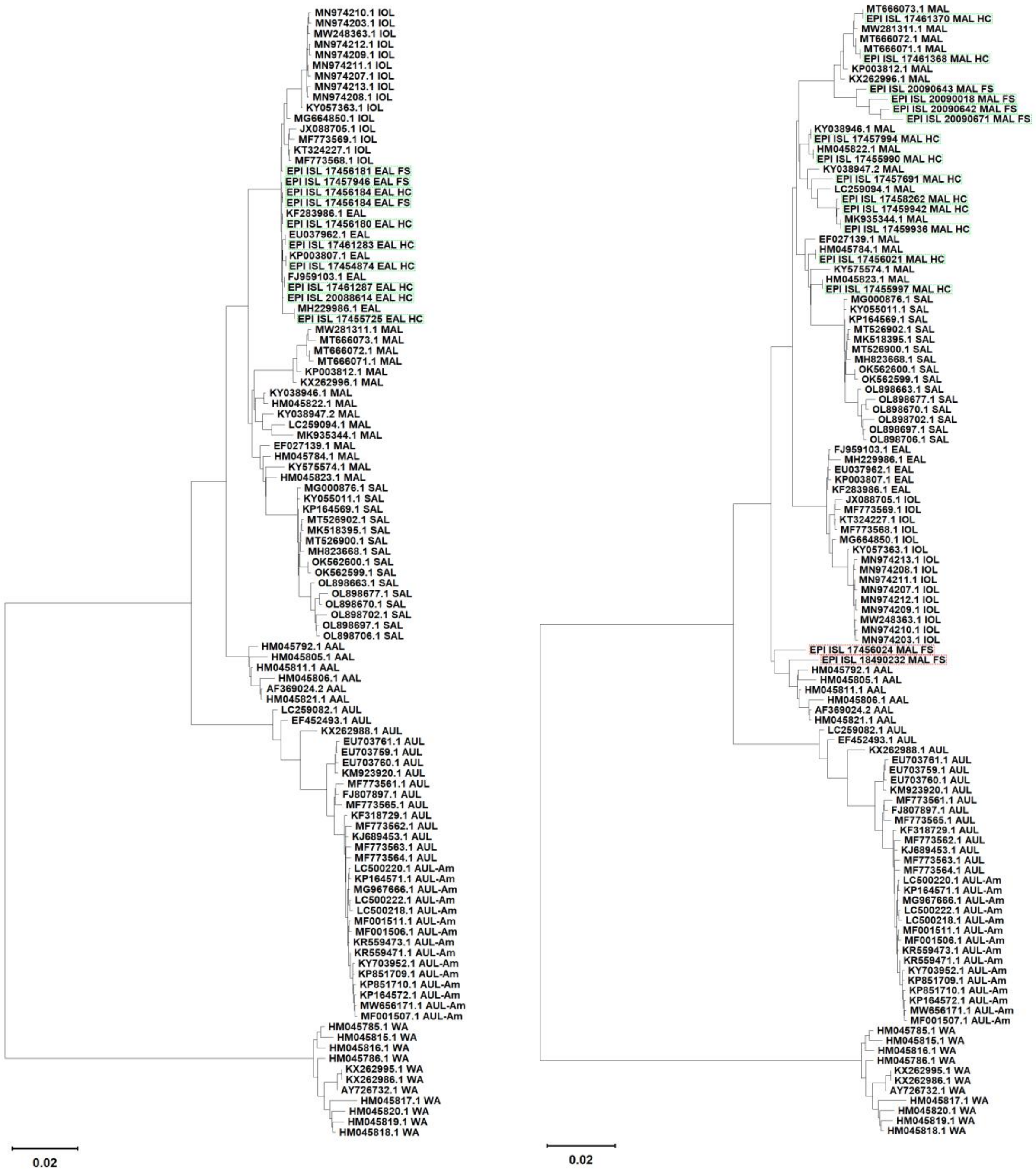

(**a**) (**b**)

Figure S2. Phylogenetic validation of ambiguous samples with two representative cases. The trees were constructed using the Maximum Likelihood method in MEGA 11. (**a**) Validation of EAL samples; (**b**) validation of MAL samples. Each panel includes high-confidence samples from the hierarchical classification (suffixed 'HC') and the ambiguous samples requiring phylogenetic validation (suffixed 'FS'). All high-confidence samples and the EAL ambiguous samples were correctly clustered. However, two GISAID-derived samples (EPI_ISL_17456024 and EPI_ISL_18490232, highlighted by red dashed boxes) originally classified as MAL by the hierarchical model were reclassified as AAL through phylogenetic analysis, demonstrating the essential role of this validation step in correcting misclassifications between closely related lineages.

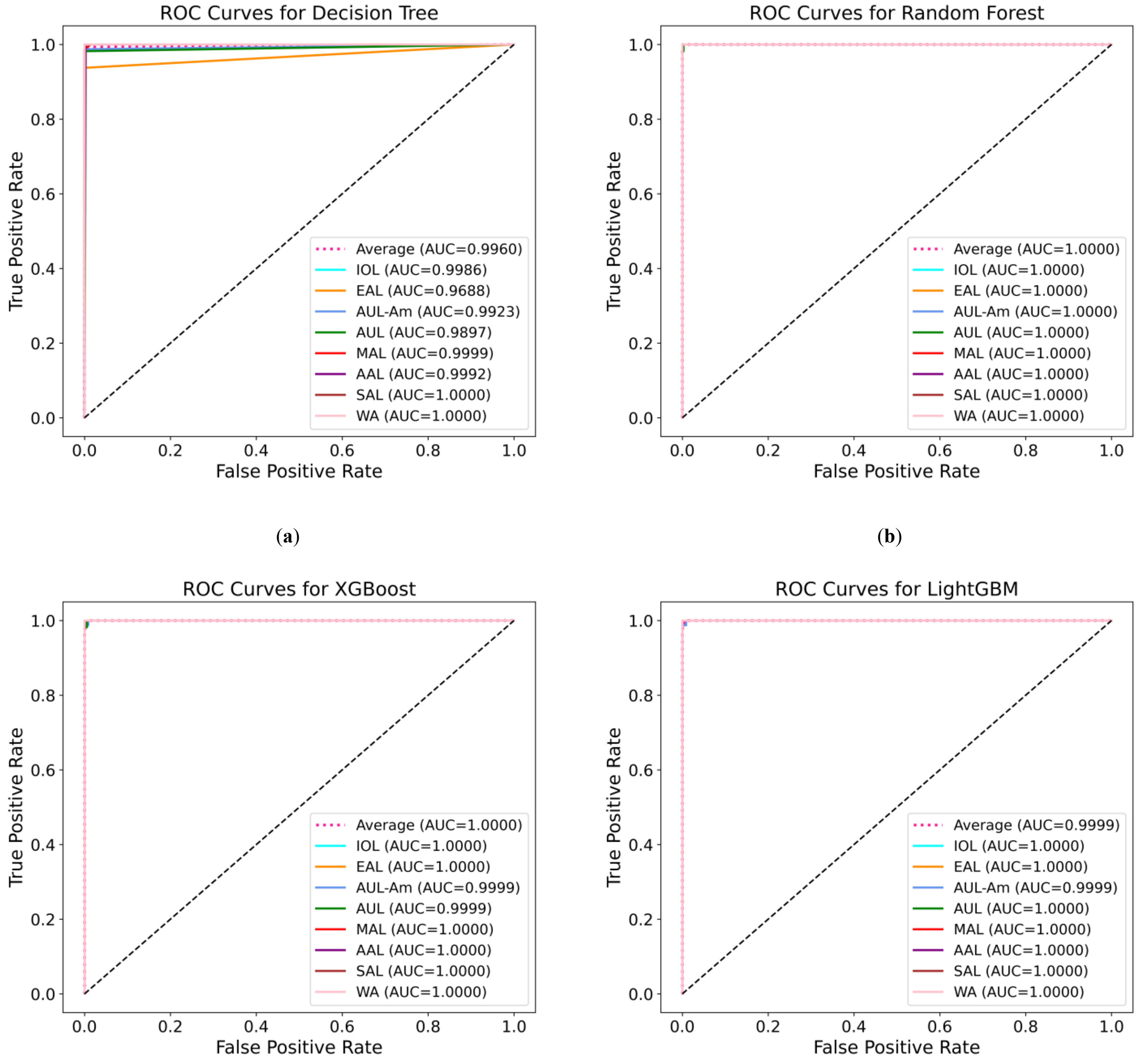

(**a**) (**b**)

(**c**) (**d**)

Figure S3. ROC curves of the nucleotide-based models on the high-coverage test set ($\mathcal{S}^{h}_{test}$). (**a**) Decision Tree; (**b**) Random Forest; (**c**) XGBoost; (**d**) LightGBM. The Area Under the Curve (AUC) value for each lineage is annotated within the plot. The pink dashed line depicts the ROC curve computed via micro-averaging, which aggregates the contributions from all lineages to estimate a global performance measure.

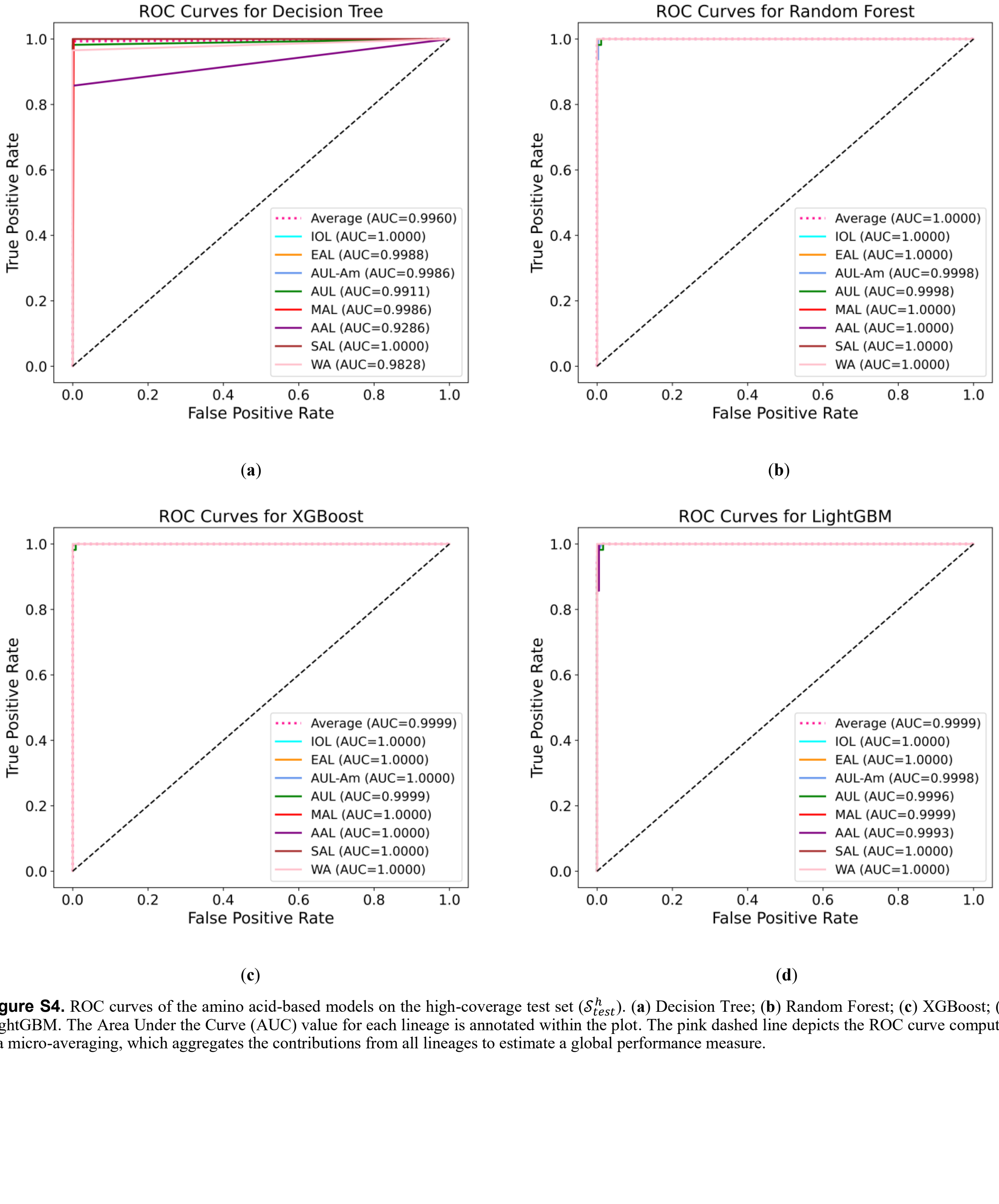

Figure S4. ROC curves of the amino acid-based models on the high-coverage test set ($\mathcal{S}^{h}_{test}$). (**a**) Decision Tree; (**b**) Random Forest; (**c**) XGBoost; (**d**) LightGBM. The Area Under the Curve (AUC) value for each lineage is annotated within the plot. The pink dashed line depicts the ROC curve computed via micro-averaging, which aggregates the contributions from all lineages to estimate a global performance measure.

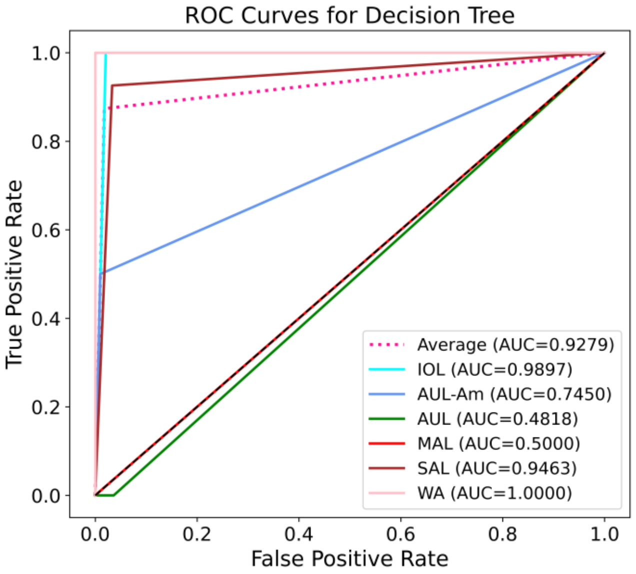

(**a**)

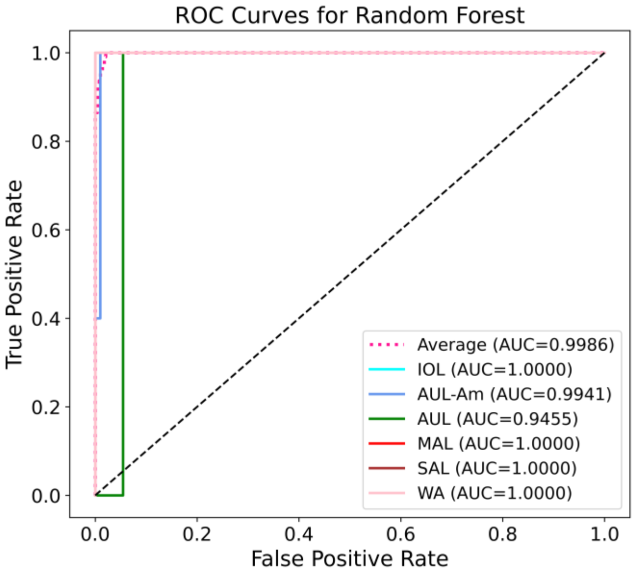

(**b**)

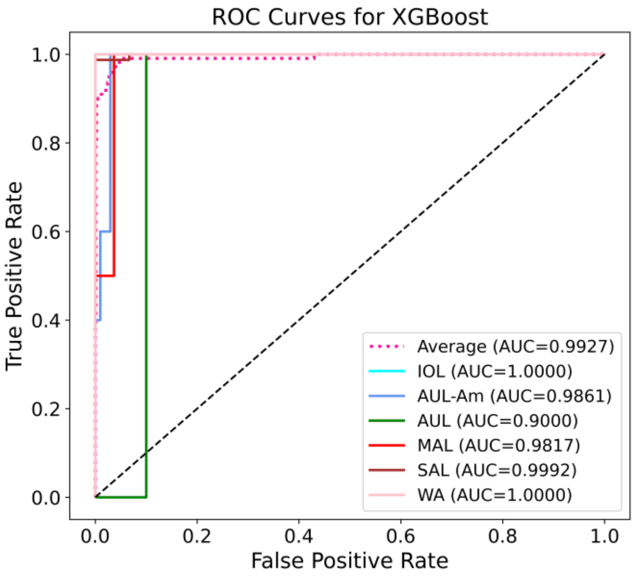

(**c**)

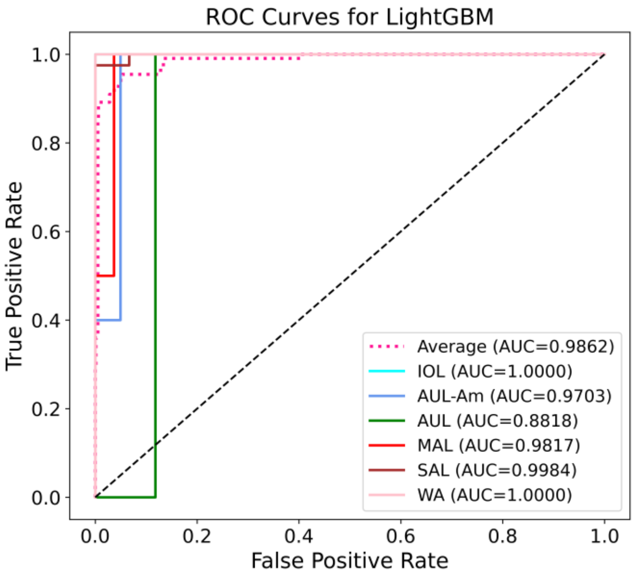

(**d**)

Figure S5. ROC curves of the nucleotide-based models on the low-coverage test set ($\mathcal{S}_{test}^{l}$). (**a**) Decision Tree; (**b**) Random Forest; (**c**) XGBoost; (**d**) LightGBM. The Area Under the Curve (AUC) value for each lineage is annotated within the plot. The pink dashed line depicts the ROC curve computed via micro-averaging, which aggregates the contributions from all lineages to estimate a global performance measure.

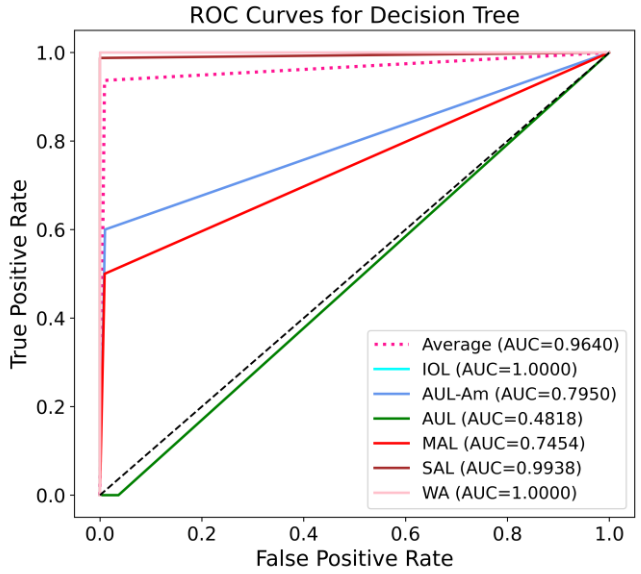

(**a**)

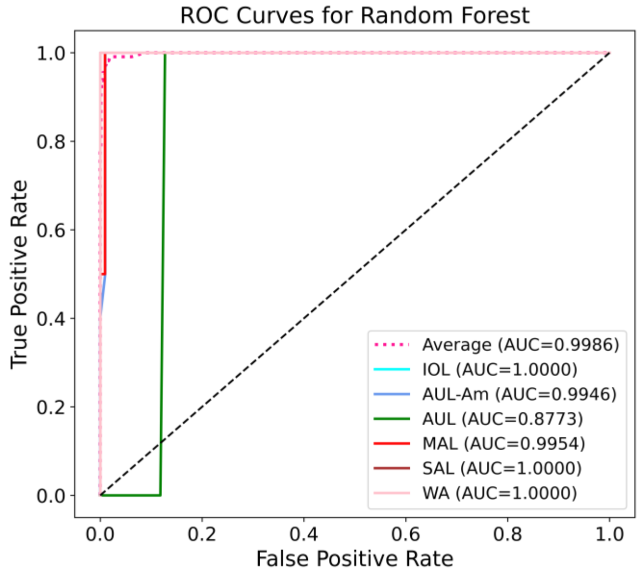

(**b**)

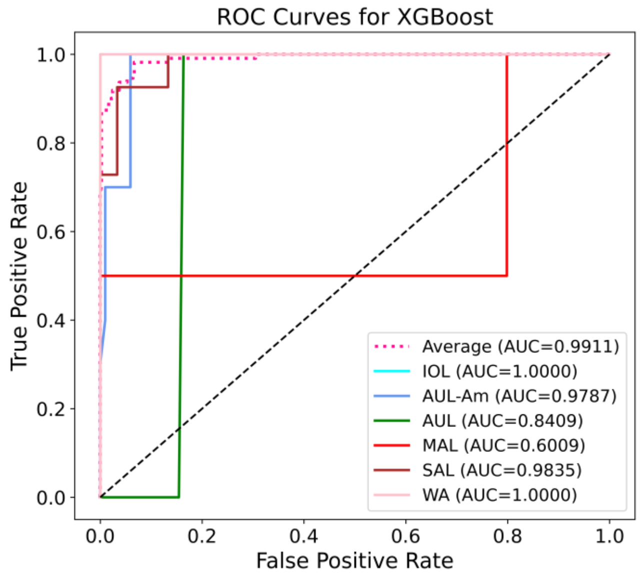

(**c**)

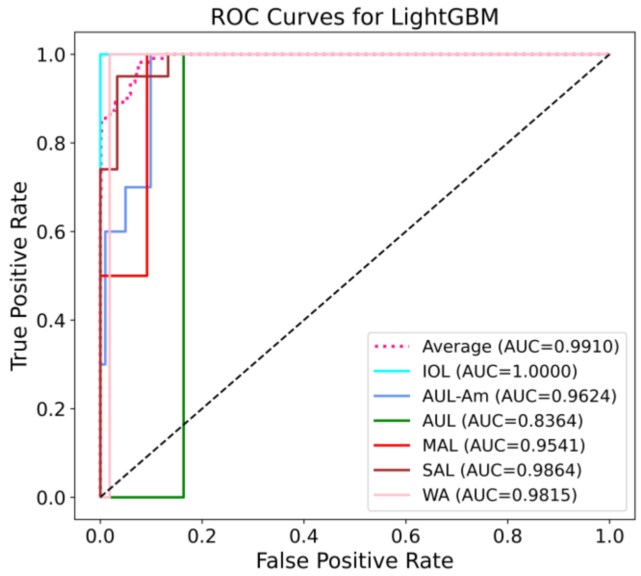

(**d**)

Figure S6. ROC curves of the amino acid-based models on the low-coverage test set ($\mathcal{S}^{l}_{test}$). (**a**) Decision Tree; (**b**) Random Forest; (**c**) XGBoost; (**d**) LightGBM. The Area Under the Curve (AUC) value for each lineage is annotated within the plot. The pink dashed line depicts the ROC curve computed via micro-averaging, which aggregates the contributions from all lineages to estimate a global performance measure.

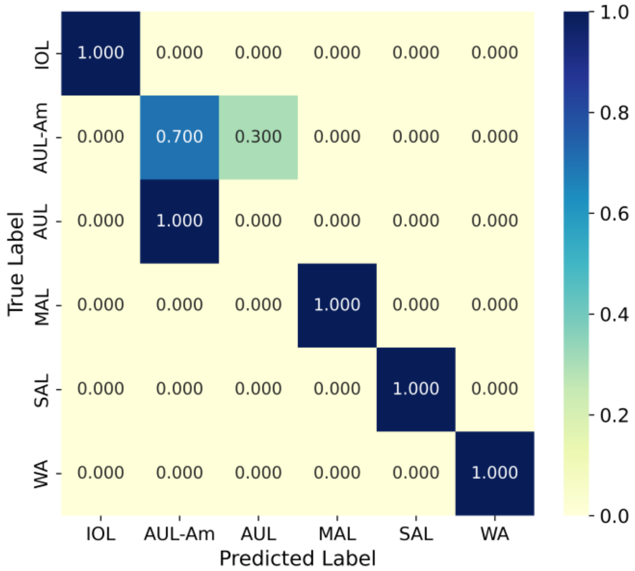

Figure S7. Confusion matrix of the optimal Random Forest classifier using amino acid-based features on the low-coverage test set ($\mathcal{S}_{test}^{l}$). The model maintains strong classification performance despite sequence incompleteness. Misclassification is limited to a portion (30%) of AUL-Am samples being assigned to AUL. Note the absence of EAL and AAL in this test set.

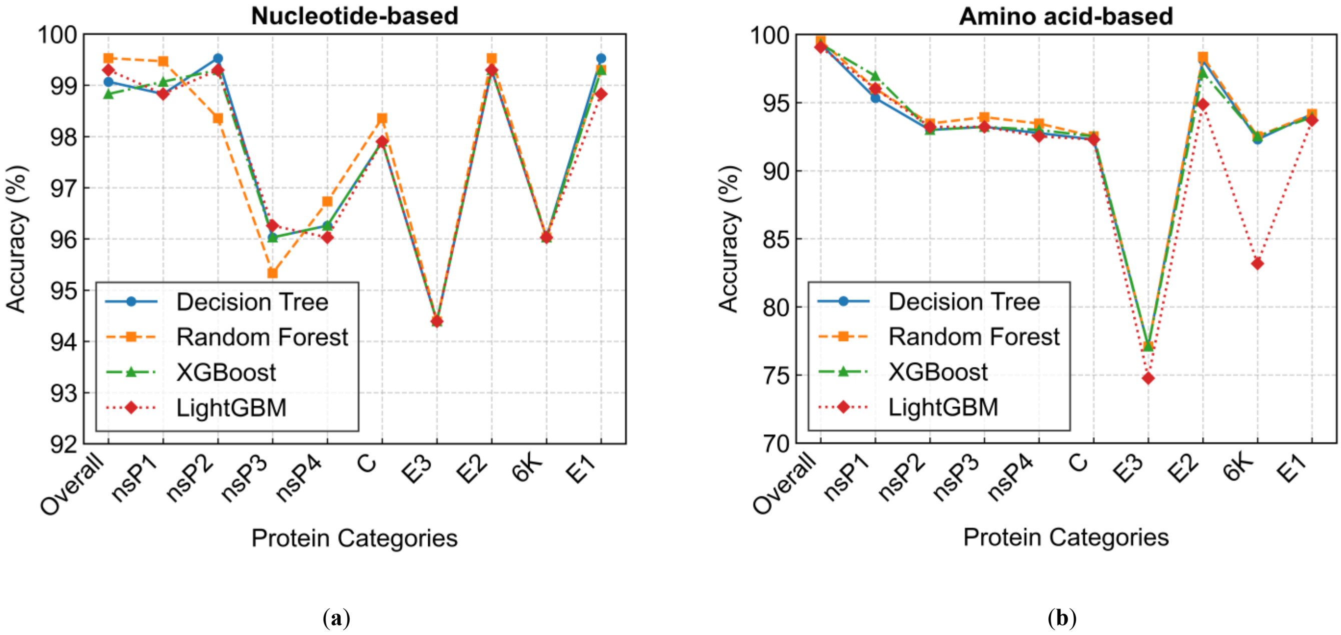

Figure S8. Performance comparison of lineage classification models trained on features from individual viral proteins in terms of accuracy. The accuracy was evaluated on the high-coverage test set ($\mathcal{S}_{test}^{h}$). (**a**) Models built on nucleotide-based features; (**b**) models built on amino acid-based features. The whole-genome model performance is included as a benchmark.

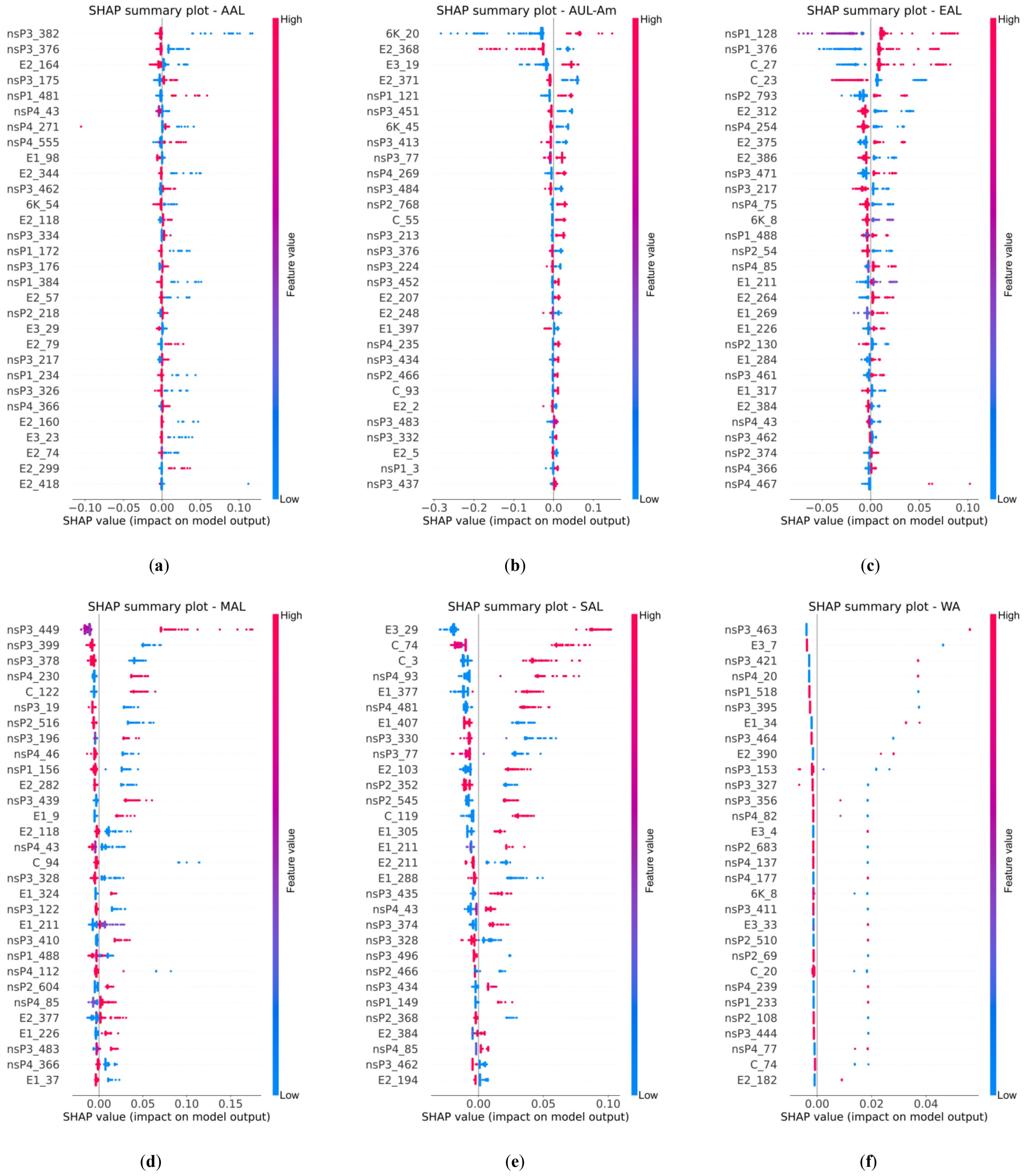

Figure S9. SHAP summary plots for six CHIKV lineages. The plots visualize the top 30 features ranked by the mean absolute SHAP value for (**a**) AAL, (**b**) AUL-Am, (**c**) EAL, (**d**) MAL, (**e**) SAL, and (**f**) WA. Each point represents a sample. The feature's impact on the model output is shown on the x-axis (SHAP value), and the features are ordered on the y-axis by importance. The color represents the amino acid identity (encoded as an integer) at that site for each sample. The SHAP value indicates the feature's impact on the prediction, and a positive SHAP value increases the probability of the sample being classified as the target lineage.

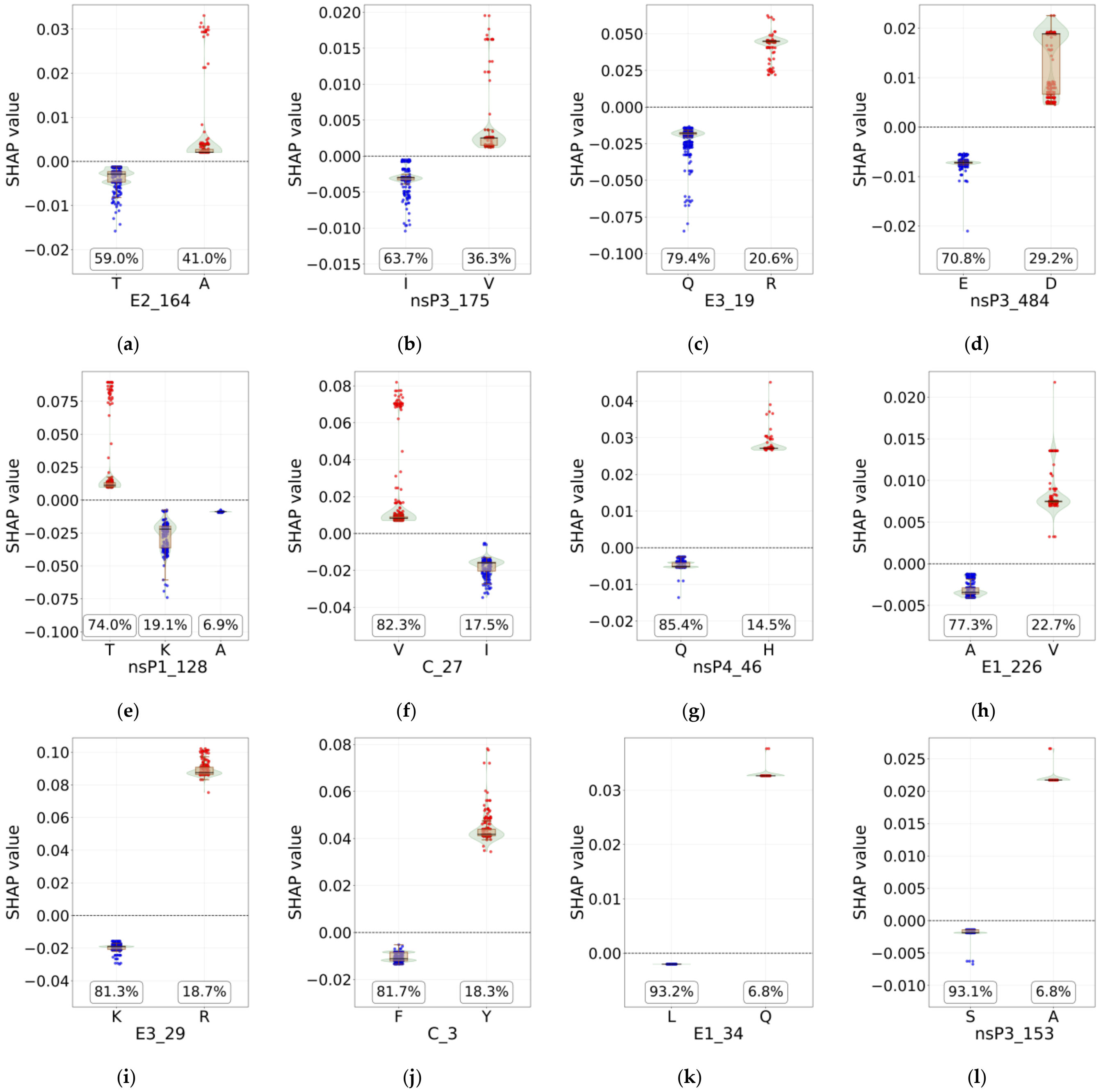

Figure S10. SHAP dependence plots for key discriminatory sites in six lineages. Two representative sites are shown for each of the following lineages: (**a**-**b**) AAL, (**c**-**d**) AUL-Am, (**e**-**f**) EAL, (**g**-**h**) MAL, (**i**-**j**) SAL, and (**k**-**l**) WA. For each site, the x-axis indicates the amino acid type, the y-axis shows the SHAP value (with a dashed line at zero), individual samples are colored by their SHAP value contribution (blue: negative, red: positive), and distributions for each amino acid are summarized with boxplots and violin plots.

Table S1. Model performance on the low-coverage test set $\mathcal{S}_{test}^{l}$.

Method	Evaluation Metrics	Feature	
		Nucleotide-based	Amino Acid-based
Decision Tree	Precision (%)	93.26	96.91
	Recall (%)	87.39	93.69
	F1-score (%)	89.82	95.10
	AUC	0.9279	0.9640
Random Forest	Precision (%)	97.60	97.97
	Recall (%)	93.69	95.50
	F1-score (%)	95.12	96.50
	AUC	0.9986	0.9986
XGBoost	Precision (%)	96.25	92.13
	Recall (%)	90.09	89.19
	F1-score (%)	92.26	90.28
	AUC	0.9927	0.9911
LightGBM	Precision (%)	96.40	89.63
	Recall (%)	89.19	86.49
	F1-score (%)	91.73	87.65
	AUC	0.9862	0.9910
